# A new European species of *Ceratophysella* (Collembola, Hypogastruridae) revealed by morphological data and DNA barcodes

**DOI:** 10.3897/zookeys.1021.63147

**Published:** 2021-03-01

**Authors:** Dariusz Skarżyński, Adrian Smolis, Ľubomír Kováč, David Porco

**Affiliations:** 1 Institute of Environmental Biology, Department of Invertebrate Biology, Evolution and Conservation, University of Wrocław, Przybyszewskiego 65, 51-148, Wrocław, Poland University of Wrocław Wrocław Poland; 2 Department of Zoology, Institute of Biology and Ecology, Faculty of Science, Pavol Jozef Šafárik University, Moyzesova 11, 041 54, Košice, Slovakia Pavol Jozef Šafárik University Košice Slovakia; 3 Musée National d’Histoire Naturelle, 25 rue Munster, 2160, Luxembourg, Luxembourg Musée National d’Histoire Naturelle Luxembourg Luxembourg

**Keywords:** Springtails, integrative taxonomy, COI sequences, *Ceratophysella stachi* sp. nov., *Ceratophysella granulata*

## Abstract

A new species, *Ceratophysella stachi*, from Denmark, Germany, Luxembourg, Norway, Poland, and Ukraine is described based on morphological data and DNA barcodes. It belongs to a small European group of species with type B chaetotaxy and strong tegumentary granulation with distinct fields of coarse granules: *C. granulata* Stach, 1949, *C. lawrencei* (Gisin, 1963), *C. neomeridionalis* (Nosek & Červek, 1970), *C. scotica* (Carpenter & Evans, 1899), and *C. silvatica* Rusek, 1964. It differs from all of them in the chaetotaxy of lateral parts of thoracic terga II–III (setae m_6_ present and one additional seta outside lateral sensillum m_7_ present or absent) that is exceptional within the whole *C. armata*-group. Notes on closely related species *C. granulata* are also given.

## Introduction

*Ceratophysella* Börner, 1932, comprising 140 species (Bellinger et al. 1996–2021), is one of the largest collembolan genera within the family Hypogastruridae. Although the genus is considered cosmopolitan, the vast majority of species live in the temperate climatic zone of the northern hemisphere. Unfortunately, some of these species are insufficiently known, and there are doubts concerning their taxonomic status. One of these is *Ceratophysella granulata* Stach, 1949. This species was described from the Tatra Mountains (Polish Carpathians) by Stach (1949) and also reported by him from Slovakia, Ukraine, the former Yugoslavia (Slovenia), and France. Then, it has been frequently recorded from various European countries: Austria (Christian 1987), Bosnia and Herzegovina (Bogojevič 1968), Denmark (Fjellberg 1998), Germany (Eckert and Palissa 1999), Great Britain (Goto 1955a, b), Hungary (Danyi and Traser 2008), Norway (Fjellberg 1998), Poland (Stach 1949, 1964, Weiner 1981, Sterzyńska and Kaprus’ 2000, Smolis and Skarżyński 2003, 2006), Romania (Danyi et al. 2006, Popa 2012), Slovakia (Nosek 1958, 1969, Kováč et al. 2016), the former Soviet Union (Grinbergs 1960), Switzerland (Gisin 1949), and Ukraine (Kaprus’ et al. 2006). However, the reliability of these data is questioned. For example, Hopkin (2007) found that British specimens from the collection of Natural History Museum in London refer to *Ceratophysella denticulata* (Bagnall, 1941). Babenko et al. (1994) came to a similar conclusion after examination of specimens identified as *C. granulata* from the area of the former Soviet Union. They found that most of them referred to other species, usually of the *C. denticulata* group. Moreover, the comparison of the morphology of the topotypic population (Skarżyński 2004a) and the populations from Denmark and Norway (Fjellberg 1998) showed subtle differences in chaetotaxy, which indicates that *C. granulata* may be a complex of species. In order to establish the taxonomic status of the forms included in this complex, a classical taxonomic analysis of materials identified as *C. granulata* from several European scientific collections and DNA barcoding were performed.

## Material and methods

### Species/populations studied

Morphological analysis of available specimens designated as *C. granulata* (C. cf. granulata, *Hypogastrura granulata*) from Denmark, Germany, Hungary, Luxembourg, Norway, Poland (including syntypes and topotypes), Romania, Slovakia, Switzerland, and Ukraine from the collections of eight institutions (Table 1) was performed. Unfortunately, specimens of *C. granulata* mentioned in the original description (Stach 1949) from Slovakia (Podlužany, orig. “Dobó–Berekalja”), West Ukraine (Czarnohora Range: Zaroślak and Breskuł), former Yugoslavia (Slovenia, Škocjan Caves, orig. “St. Canzian cave”), and France (Arles) could not be found in the ISEZ collection (*in litt*. Wanda M. Weiner); therefore, they were not examined. The search for materials of this species mentioned in the “Catalogus faunae Austriae” (Christian 1987) in the collection of Natural History Museum Vienna also did not bring any results (*in litt*. Harald Bruckner). In addition, the sequences from 21 specimens from five species were analysed to assess the status of *C. granulata* forms in the context of the genetic divergence within the genus (Table 2).

**Table 1.** A list of institutions and countries in which specimens are deposited, with abbreviations.

Abbreviation	Depository	Country
AF	Collection of Dr Arne Fjellberg	Norway
DIBEC	Department of Invertebrate Biology, Evolution and Conservation, University of Wrocław	Poland
PJSU	Department of Zoology, Institute of Biology and Ecology, Faculty of Science, Pavol Jozef Šafárik University, Košice	Slovakia
HNHM	Hungarian Natural History Museum, Budapest	Hungary
ISEZ	Institute of Systematics and Evolution of Animals, Polish Academy of Sciences, Cracow	Poland
MHNG	Muséum d’histoire naturelle, Geneva	Switzerland
SMNG	Senckenberg Museum of Natural History, Görlitz	Germany
SMNHL	State Museum of Natural History, Ukrainian National Academy of Sciences, L’viv	Ukraine

**Table 2.** List of barcoded species. *N* = number of sequences available.

Species	Collecting data (all from Poland)	*N*	Published sequences
*Ceratophysella granulata* (= *C. stachi* sp. nov.)	Beskid Niski Mountains, Carpathians, litter of the Carpathian beech forest on the slopes of Ostra Góra near village Tylawa, at an altitude of 500 m a.s.l., 20.X.2009, leg. M. Furgoł	2	–
*Ceratophysella granulata*	Tatra Mountains, Carpathians, litter of dwarf mountain pine shrubs on the slopes of the Gładkie Upłaziańskie, at an altitude of 1600 m a.s.l., 14.VIII.2009, leg. D. Skarżyński	5	Porco et al. (2012)
*Ceratophysella denticulata*	Nizina Śląska Lowland, oak-hornbeam forest in Wrocław, 10.X.2009, leg. D. Skarżyński	5	–
*Ceratophysella cavicola*	Karkonosze Mountains, Sudetes, old adit Krucze Skały near Karpacz, 650 m a.s.l, 6.VI.2009, leg. D. Skarżyński	4	Porco et al. (2012)
*Ceratophysella engadinensis*	Wzgórza Trzebnickie Hills, peat bog near Twardogóra, 11.X.2009, leg. D. Skarżyński	5	–

### Morphology

Specimens stored in alcohol were cleared in Nesbitt’s fluid (chloral hydrate, concentrated hydrochloric acid, distilled water), slide-mounted in a mixed medium (distilled water, gum arabic, glycerol, chloral hydrate), and studied using a Nikon Eclipse E600 phase contrast microscope. Figures were drawn using a camera lucida. A set of characters commonly used in the taxonomy of the genus (Fjellberg 1984, 1998, 1999; Babenko et al. 1994; Thibaud et al. 2004) was analysed.

### DNA barcoding

Lysis of the tissues was carried out in 50 µl volume of lysis buffer and proteinase K incubated at 56 °C overnight. DNA extraction followed a standard automated protocol on 96-well glass fibre plates (Ivanova et al. 2006), and during this DNA extraction, a voucher recovery specially designed for high-throughput workflow (Porco et al. 2010) was used. The 5' region of COI used as a standard DNA barcode was amplified using M13 tailed primers LCO1490 and HCO2198 (Folmer et al. 1994). A standard PCR reaction protocol was used for PCR amplifications and products were checked on a 2% E-gel 96Agarose (Invitrogen). Unpurified PCR amplicons were sequenced in both directions using M13 tails as primers. The sequencing reactions followed standard protocols of the Canadian Centre for DNA Barcoding (Hajibabaei et al. 2005), with products subsequently purified using Agencourt CleanSEQ protocol (Agencourt) and processed using BigDye v. 3.1 on an ABI 3730 DNA Analyzer (Applied Biosystems). Sequences were assembled with Sequencer v. 4.5 (GeneCode Corporation, Ann Arbor, MI, USA) and aligned by eye using BIOEDIT v. 7.0.5.3 (Hall 1999); we observed no indels in this coding region of the mitochondrial genome, and therefore, all base positions were aligned with confidence in positional homology. Sequences are publicly available on BOLD (Ratnasingham and Hebert 2007; http://www.barcodinglife.org) within the public dataset NCERAT and in GenBank (HM398990–HM399010, JX261875, MW471668). Distance analyses were conducted with MEGA7 (Tamura et al. 2007) using a Neighbor-Joining (Saitou and Nei 1987) algorithm and distances corrected with the Kimura-2 parameter (Kimura 1980). Kimura-2 parameter is the best metric when distances are low (Nei and Kumar 2000). The robustness of nodes was evaluated through bootstrap re-analysis of 1000 pseudoreplicates.

## Results

### Morphology

The material under study appeared to be taxonomically heterogeneous. Hungarian specimens (HNHM, “*Hypogastrura granulata*” det. Loksa: 27 spp., Sz(N.) 1975/76, Szentbékkála, 1975.05.09, leg. Loksa, coll-1868 and 2 spp., Bátorliget, 1989-90, coll-1008) were identified as *C. denticulata* and *C. silvatica* (Rusek, 1964). Most of the specimens from Ukraine (SMNHL, “*Ceratophysella granulata*”, Carpathians: 5 spp., Perkalab river, litter of spruce forest, 1.VIII.1991, leg. I. Kaprus’, 2.2.4.5; 2 spp., Skole, litter of beech forest, 3.IV.2004, leg. Javornitsskij, 2.2.4.4; 2 spp., Vorokhta, litter of spruce forest, 5.IX.1999, leg. Javornitsskij, 2.2.4.7; 2 spp., Borzhava, 1200 m a.s.l, soil and turf, 5.XI.1996, leg. L. Sukovata, 2.2.4.6) appeared to be the epitokous form of *C. silvatica*, while Swiss specimens suitable for examinaton (MHNG, Gisin’s collection, “Hypogastrura cf. granulata”: 2 spp., Neuchâtel, Forêt de Chuffort, Mont Chaumont, 8 km from Neuchâtel; ca 1170 m, X.1963, Fn 51; 14 spp., Genève, Vessy, forest, ca 400 m, samples from an ecological study in 1946–1948, Ga57) were assigned to *C. armata* (Nicolet, 1842) and *C. attenuata* Cassagnau, 1959. The original designations of some specimens from Luxembourg also proved to be erroneous, six specimens from Obereisenbach (5 juv., Holzbichsbaach, oaks, litter and mosses, 5.VII.1991, leg. Tommasi-Ursone, L-91-51 and 1 juv., Husterbaach, beeches, in nettles, *Geranium*, *Digitalis*, etc., 5.VII, 1991, leg. M. Ursone, L-91-53, ISEZ) actually belong to *C. denticulata*. An unambiguous determination of species status of juvenile individuals with type B chaetotaxy and integument with fields of especially coarse granules from three samples (1 juv., Poland, Puszcza Zgorzelecka, Ruszów, temperate thickets and scrub, *Spiraea* site, mowed, 16.X.2013, leg. U. Burkhardt, 882-01; 5 juv., Germany, Saxony, Zittau, Roschertal, Mandau river valley near Hainewalde, sample 27, mosses on meadow, 10 m above Mandau, 3.V.1972, leg. W. Dunger, 8825-8827, SMNG and 1 juv.; Romania, Carpathians, Maramureş District, Rodnei Mts, Borşa, N slope of Pietrosul Rodnei, ca 1700 m a.s.l,, 27.VII.2004, leg, J. Radwański, RU/04/1/70, ISEZ) was unsuccessful due to the lack of useful features distinguishing immature stages.

**Figure 1.**
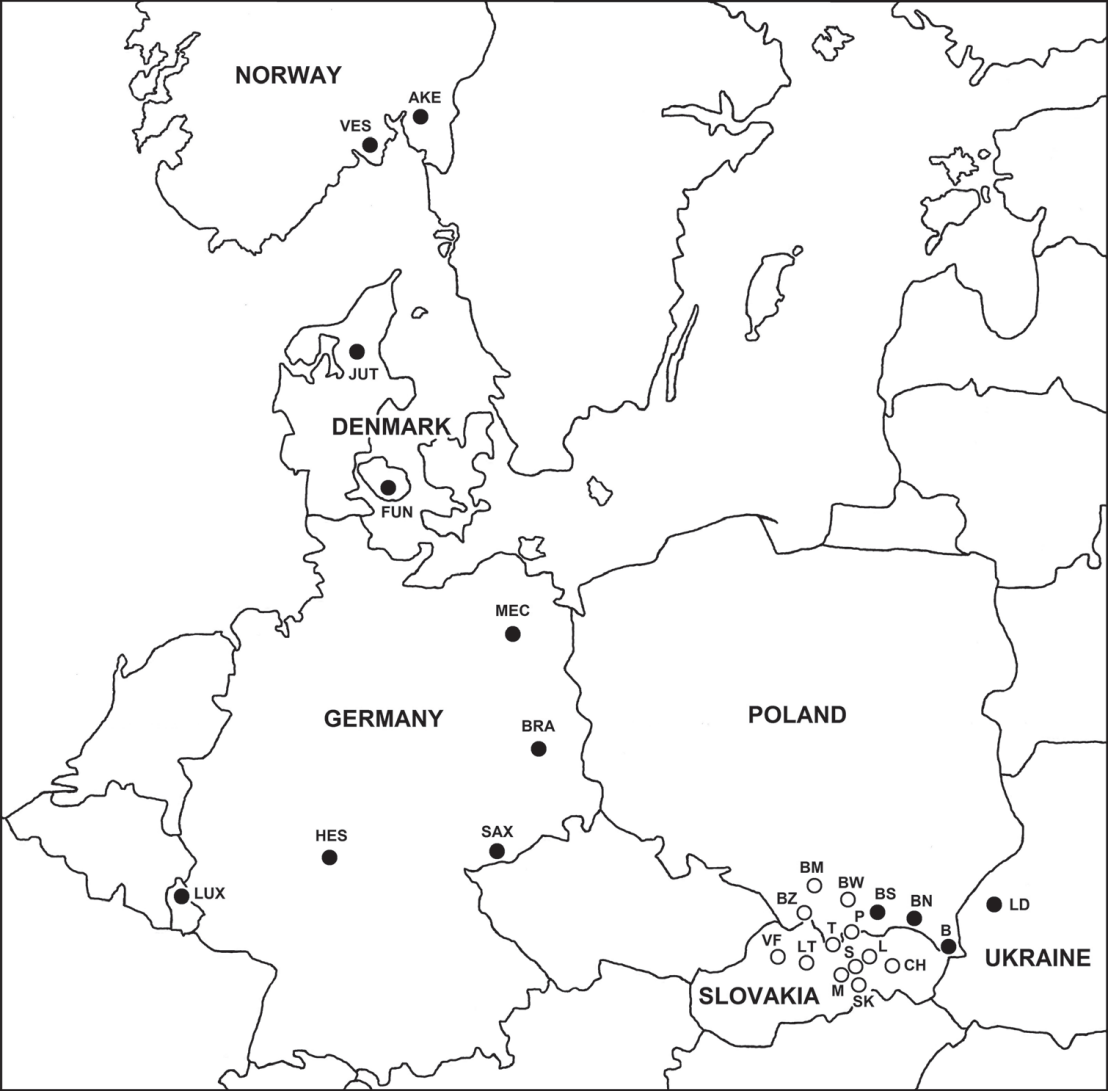
Distribution of *Ceratophysella stachi* sp. nov. (black circles) and *C. granulata* (empty circles). Abbreviations: AKE – Akershus, B – Bieszczady Mts, BM – Beskid Mały Mts, BN – Beskid Niski Mts, BRA – Brandenburg, BS – Beskid Sądecki Mts, BW – Beskid Wyspowy Mts, BZ – Beskid Żywiecki Mts, CH - Čierna hora Mts, FUN – Funen, HES – Hesse, JUT – Jutland, L - Levočské vrchy Mts, LD – Lviv District, LT – Low Tatra Mts, LUX – Luxembourg, M – Muránska planina Plateau, MEC – Mecklenburg-West Pomerania, P – Pieniny Mts, S – Slovak Paradise, SAX – Saxony, SK – Slovak Karst, T – Tatra Mts, VES – Vestfold, VF – Veľká Fatra Mts.

Among the examined specimens that can be referred to *C. granulata*, two morphotypes were found, differing in the chaetotaxy of the lateral part of thoracic terga II–III, the size and shape of accessory boss near the postantennal organ, and the shape of mucro. Considering their clear morphological differentiation, both forms are treated as separate species. Thus, the form from the northwestern part of the Carpathians (Poland and Slovakia) was recognized (based on syntypes and topotypes) as *C. granulata*, whereas the form distributed in Norway through Denmark, Germany, Luxembourg, and the eastern part of Polish Carpathians to Ukraine (Fig. 1) was considered to be a species new to science–*C. stachi* sp. nov. Notes on the former and description of the later are given below.

### DNA barcoding

The mean genetic divergence among the four *Ceratophysella* species included into the analysis was 24.2% (ranging from 19% to 28.2%), and their mean intraspecific variation was 0.5% (ranging from 0% to 0.6%). Similar values were found for both interspecific (mean 23% ranging from 21% to 24.4%) and intraspecific (1%) in *C. stachi* sp. nov., thus supporting the status of the new species (Table 3, Fig. 2). Moreover, these values are in line with the usual ‘barcoding gap’ described so far in the family Hypogastruridae (e.g. Nakamori 2013; Skarżyński et al. 2018) but also more generally in Collembola (e.g. Porco et al. 2014; Nilsai et al. 2017).

**Table 3.** Intraspecific and interspecific % of K2P distances in the targeted *Ceratophysella* species.

#	Species	Intraspecific distances	Interspecifc distances				
			1	2	3	4	5
1	*C. cavicola*	0.2					
2	*C. denticulata*	0.6	26.6				
3	*C. engadinensis*	0.0	28.2	19.0			
4	*C. granulata*	1.1	21.3	24.3	25.9		
5	*C. stachi* sp. nov.	1.0	24.4	23.6	22.9	21.0	–

**Figure 2.**
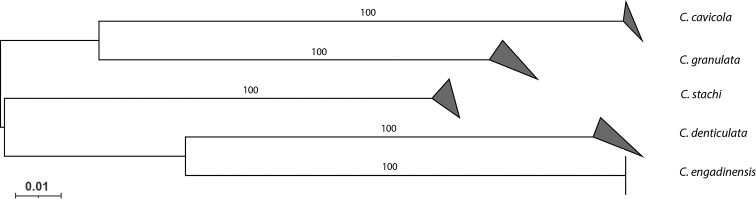
Neighbor joining tree (K2P) of the five *Ceratophysella* species targeted (based on the 5' end of COI). Bootstrap supports showed on the branches. The upper and lower side of the triangle represent respectively the maximum and minimum of genetic distances within the species.

### Taxonomy

#### Ceratophysella stachi

Taxon classificationAnimaliaPoduromorphaHypogastruridae

Skarżyński, Smolis & Porco sp. nov.

30489097-9441-5CAF-AF77-12BC8C024C10

http://zoobank.org/40BF0CC2-AF12-43DB-9F35-368E6EC004F8

Figures 3–4 , 5–9

Ceratophysella granulata : Fjellberg, 1998: 41.

##### Type material.

***Holotype* (DIBEC)**: female, Poland, Carpathians, Beskid Niski Mts, litter of the Carpathian beech forest on the slopes of Ostra Góra near Tylawa village, 500 m a.s.l., 20.X.2009, leg. M. Furgoł. ***Paratypes*** (**DIBEC)**: 2 males, same data as holotype; male, juv., 14.V.2001, leg. A. Smolis, D. Skarżyński, other data same as holotype; 3 females, 5 males, juv., 14.V.2002, leg. A. Smolis, D. Skarżyński, other data same as holotype.

##### Other material.

Denmark (AF, leg. A. Fjellberg) : female, Jutland, Himmerland, Rold Skov, *Fagus* liter, 20.III.1994, 94.024; Funen: 6 females, 2 males, Fiskerup Skov, forest stream, 24.III.1994, 94.076; female, Syltemade Adal, *Fraxinus*/*Ulmus*/*Viburnum* litter, 23.III.1994, 94.070. Germany (SMNG): Brandenburg: 6 females, 3 males, 3 juv., “Wanninchen” nature reserve, bog, 1.V.1972, leg. Hiebsch, 11911; 9 females, male, “Wanninchen” nature reserve, wet heather, 1972, leg. Hiebsch, 11911; male, 2 juv., “Bergener Moor” nature reserve, moor, *Sphagnum*, heather, pine forest, 1.V.1972, leg. Hiebsch, 11912. Hesse: 5 females, Vogelsberg, 1985–1995, leg. W. Böhle. Mecklenburg-West Pomerania: female, Müritz National Park, Neustrelitz, soil 0–5 cm, alder swamp woods not on acid peat, 16.X.2013, leg. U. Burkhardt, 264-F865; 4 females, 2 males, Serrahn, Hauptmannsberg near Feldberg, sandy-gravelly moraine, largely unforested since the Middle Ages, 1973, leg. Hiebsch/ILN Greifswald, 11915; female, Serrahn, Klockenbruch, active, relatively undamaged raised bogs, moss/Sphagnum, 16.X.2013, leg. R. Lehmitz and U. Burkardt, 261-01; male, Serrahn, Klockenbruch, active, relatively undamaged raised bogs, moss/Sphagnum, 5.XI.2014, leg. R. Lehmitz and U. Burkardt, 401-12; female, Serrahn, “Kesselmoor” nature reserve, active, relatively undamaged raised bogs, moss/Sphagnum, 16.X.2013, leg. R. Lehmitz, 49077-08; male, Serrahn, soil 0–5 cm, Medio-European collinear woodrush beech forest, 16.X.2013, leg. U. Burkhardt, 263-03. Saxony: female and juv., Erzgebirge, Kleiner Kranichsee, *Sphagnum*, 22.VII. 1971, leg. W. Dunger, 7871. Luxembourg, subadult male, Vallée d’Our: Tintesmühle, near the river, 21.VII.1991, leg. M. Ursone, L-91-101 (ISEZ). Norway (AF, leg. A. Fjellberg): Akershus: 4 females, “Östmarka” nature reserve, Tappenbergvann, old spruce cones, 18.V.1995, 95.163; female, Barum, Dalivannet, 16.XI.1997. Vestfold: male, 32/93; 2 females, Larvik, N. Holtesetra, Hvarnes, lush *Alnus* and *Fraxinus* forest, 24.XI.2007, 7.290; female and male, Larvik, Granasnekollen, Hvarnes, litter, oak/beech, 25.IX.2004, 04.086; 3 females, Brunlanes, Hummerbakken, Telemark Camp., plant debris, beach, 14.XI.1993, 93.077; female, Tjøme, Sandø, N-stranda, spongy *Pinus* litter behind beach, 22.IV.2009, 9.086. Poland (Carpathians, DIBEC): female, 3 males, 2 juv., Beskid Sądecki Mts: “Las Lipowy Obrożyska” nature reserve near Muszyna, 600 m a.s.l., mosses on rocks and trees, 1.V.2004, 25.VI.2005, leg. A. Smolis, D. Skarżyński; female, Roztoka Ryterska, 600 m a.s.l., litter and mosses near stream, 3.V.2004, leg. A. Smolis, D. Skarżyński; 5 females, “Uhryń” nature reserve, 850 m a.s.l., litter of fir-beech forest, 3.V.2000, leg. A. Smolis; female, 3 males, 2 juv., “Barnowiec” nature reserve, 850 m a.s.l., mosses on rocks in an old beech forest, 10.V.2003, leg. A. Smolis, D. Skarżyński; 4 females, 2 males, SE slopes of Jaworzyna Krynicka, litter in a beech forest, ca 800 m a.s.l, 2.V.2004, leg. A. Smolis, D. Skarżyński. Bieszczady Mts: 2 females, N slopes of Krzemieniec, 1000 m a.s.l., litter in a stream valley, 19.V.2000, leg. A. Smolis. Ukraine: male, Ivano-Frankove village, Lviv District, beech and elm forest, leaf litter and soil, leg. S. Bakaeva, 2.2.4.8 (SMNHL).

##### Etymology.

Dedicated to Jan Stach, the excellent specialist in Collembola.

##### Description.

**Figures 3, 4.**
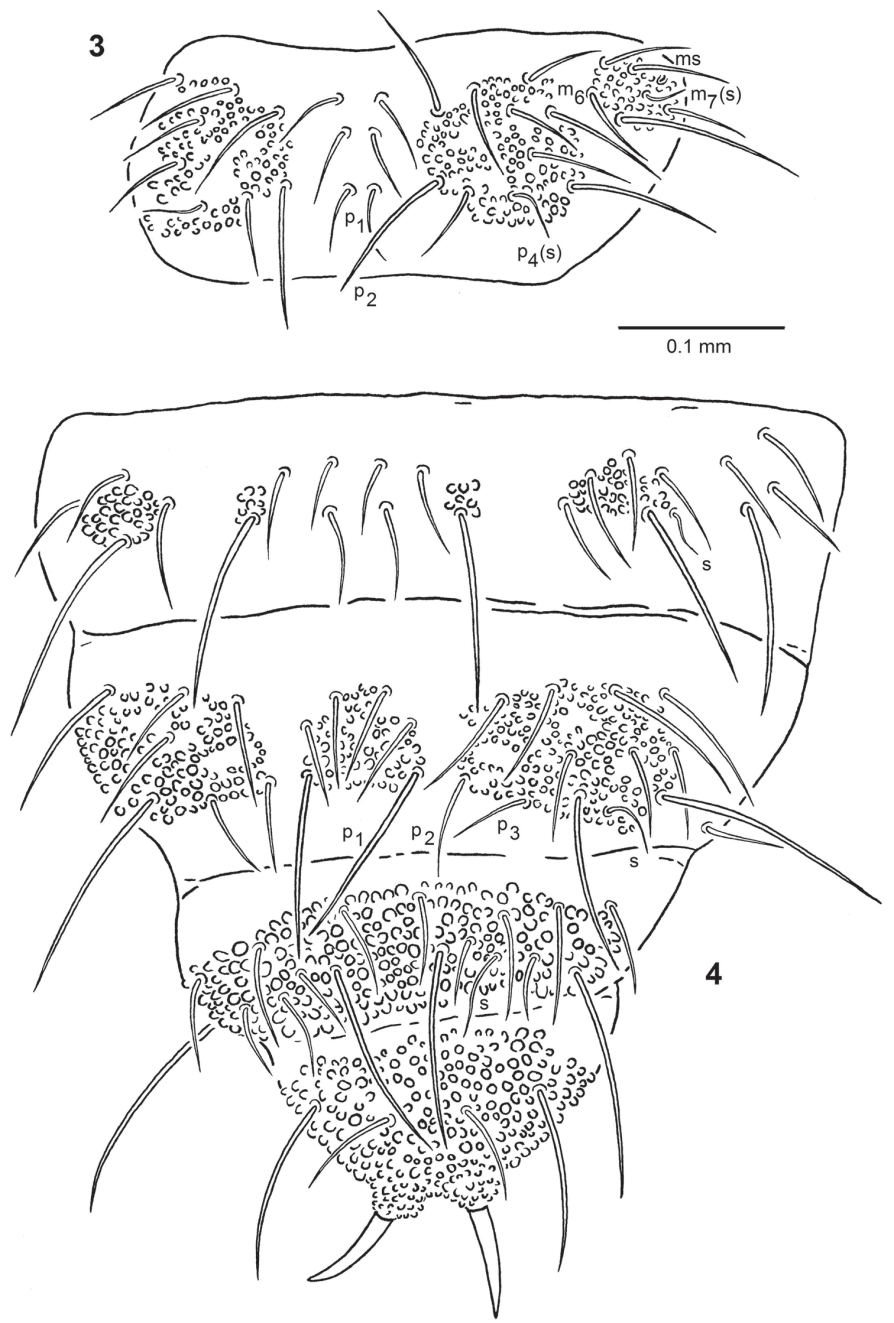
*Ceratophysella stachi* sp. nov. **3** chaetotaxy of thoracic tergum II **4** chaetotaxy of abdominal terga III–VI.

Body length 1–2 mm. Colour (in alcohol) bluish-gray to bluish-black. Tegumental granulation strong, with fields of especially coarse granules on head (large uniform field covering whole dorsal side except antennal bases), thoracic terga II–III (two large subaxial fields and two lateral ones of medium size, Fig. 3), abdominal terga I–III (variable distribution: from four – Fig. 4 to seven fields of medium size as in *C. granulata*; see Skarżyński 2004a: fig. 8), abdominal tergum IV (medium axial field and two lateral large ones, Fig. 4) and abdominal terga V–VI (large uniform fields covering almost whole dorsal side, Fig. 4). 6–9 granules between macrosetae p_1_ on abdominal tergum V.

Arrangement of setae on head typical for the genus, spine-like setae absent. Dorsal chaetotaxy of type B (Figs 3, 4). Thoracic terga II–III with setae m_6_ present and one additional seta outside lateral sensillum m_7_ present or absent. Setae p_1_ on abdominal tergum IV developed as macrosetae, p_2_ as microsetae, setae p_3_ present. Differentiation of dorsal setae into micro- and macrosetae distinct. Setae long (ratio p_1_ microsetae and p_2_ macrosetae on thoracic tergum II/inner edge of claws III = 1–1.3 and 1.8–2.8, respectively), thick, curved, pointed at tips and only slightly serrate. Body sensilla (s) short (ratio sensillum p_4_ and m_7_ on thoracic tergum II/inner edge of claws III = 0.6–1 and 0.4–0.6, respectively), thin and smooth. Microsensilla (ms) on thoracic tergum II present (Fig. 3). Subcoxae I, II, III with 1, 2, 3 setae, respectively.

**Figures 5–9.**
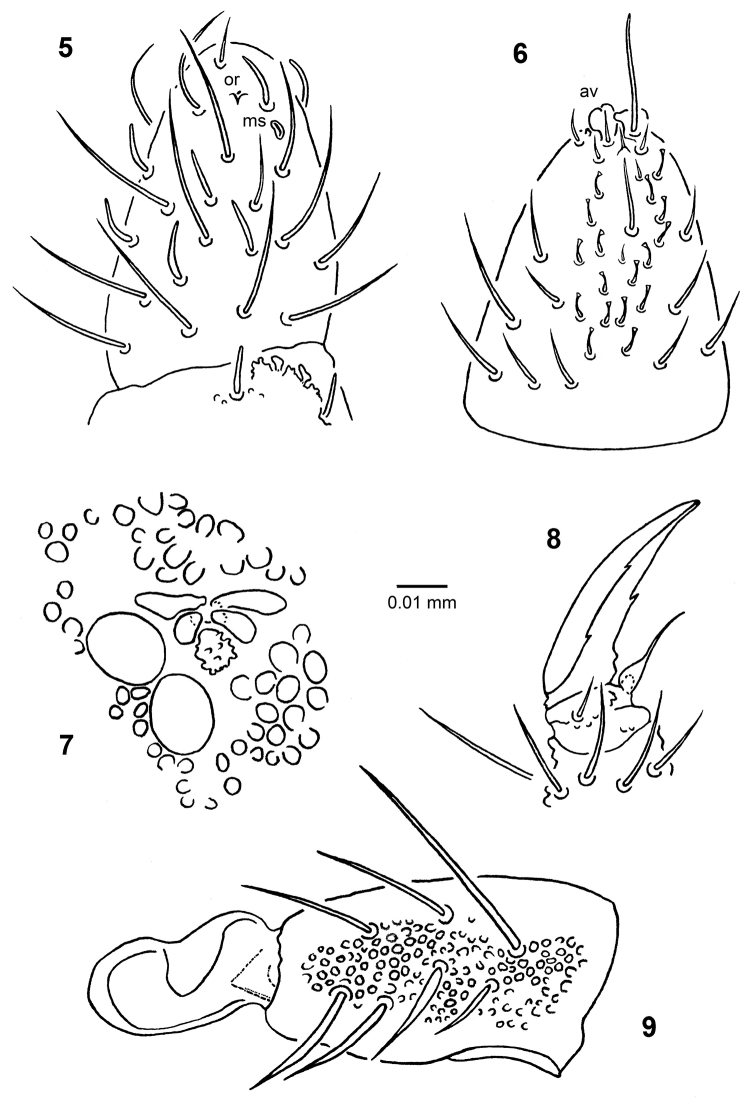
*Ceratophysella stachi* sp. nov. **5** chaetotaxy of dorsal side of antennal segments III–IV **6** chaetotaxy of ventral side of antennal segment IV **7** postantennal organ and neighbor ocelli **8** claw I **9** dens and mucro.

Antennal segment IV with simple or lobed apical vesicle, subapical organite (or), microsensillum (ms), 7 (2 lateral, 5 dorsal) cylindrical, subequal sensilla and 15–25 slightly curved blunt-tipped sensilla in ventral field (Figs 5, 6). Antennal segment III organ with two long (lateral) and two short (internal), curved sensilla (Fig. 5). Microsensillum on antennal segment III present. Eversible sac between antennal segments III and IV present. Antennal segment I with 7 setae.

Ocelli: 8 + 8. Postantennal organ 1.8–2.3 times as large as single ocellus; the former with four lobes, its anterior pair larger than posterior pair. Accessory boss large (equal to or only slightly smaller than posterior lobes of postantennal organ), often granulated (Fig. 7).

Labrum with 5, 5, 4 setae; 4 prelabrals present. Maxillary head of *C. armata* type (Fjellberg 1984: fig. 18). Labial palp as shown in Fjellberg (1999: Fig. 4), but with 6 proximal setae. Outer maxillary lobe with one sublobal hair.

Tibiotarsi I, II, III with 19, 19, 18 setae, respectively, clavate setae absent. Claws with inner tooth and a pair of lateral teeth. Empodial appendage with broad lamelliform base and filiform apex reaching inner tooth or slightly beyond, ratio empodial appendage/ inner edge of claws = 0.4–0.7 (Fig. 8).

Ventral tube with 4 + 4 setae. Furca well developed. Ratio dens + mucro/inner edge of claw III = 1.8–2.2, ratio dens/mucro = 1.7–2.2. Dens with uniform fine granules and 7 dorsal setae (2–4 inner setae modified) (Fig. 9). Mucro wide at tip (ratio width of apical part/length of mucro = 0.4–0.6, usually 0.5), boat-like, with large outer lamella, (Fig. 9). Retinaculum with 4 + 4 teeth.

Anal spines yellowish, slightly curved, situated on high basal papillae, 1.1–1.7 times as long as inner edge of claws III (Fig. 4).

##### Distribution and ecology.

The range of distribution of *C. stachi* sp. nov. appears to be relatively wide. It is known from Denmark (Jutland, Funen), Germany (Brandenburg, Hesse, Mecklenburg-West Pomerania, Saxony), Luxembourg, southern Norway (Akershus, Vestfold), Poland (Carpathians: Beskid Niski, Beskid Sądecki, Bieszczady mountains) and Ukraine (Lviv District) (Fig. 1). Probably this species is distributed much more widely in Europe, but additional research is needed to prove this thesis. *Ceratophysella stachi* sp. nov. lives in lowlands and in the mountains (up to ca. 1000 m a.s.l) where it inhabits litter and mosses in different types of forests, and also heathlands and bogs.

##### Remarks.

*Ceratophysella stachi* sp. nov. belongs to a small European branch of species of the *C. armata*-group, which have strong tegumentary granulation, with distinct fields of coarse granules: *C. granulata*, *C. lawrencei* (Gisin, 1963), *C. neomeridionalis* (Nosek & Červek, 1970), *C. scotica* (Carpenter & Evans, 1899), and *C. silvatica*. It differs from all of them in the chaetotaxy of the lateral parts of the thoracic terga II–III (setae m_6_ present and one additional seta outside lateral sensillum m_7_ present or absent vs setae m_6_ and additional setae absent) which is exceptional within the whole *C. armata*-group (both characters are found in the genus, but in other groups of species: formerly classified as *Mitchellania* Wray, 1953 and *C. denticulata*). The remaining differences between *C. stachi* sp. nov. and related species mentioned above are summarized in Table 4.

**Table 4.** Characteristics of *Ceratophysella stachi* sp. nov., *C. granulata* and related species. Based on Carpenter and Evans (1899), Gisin (1963), Rusek (1964), Nosek and Červek (1967, 1970), Christian (1987), Babenko et al. (1994), Fjellberg (1998), Skarżyński (2004a, 2006), Thibaud et al. (2004), Kaprus’ et al. (2006), Danyi and Traser (2008), Kováč et al. (2016), and our own data. Abbreviations: d_2_ - spine-like setae d_2_ on head, oc_2_ – spine-like setae oc_2_ on head, m_6_ – setae m_6_ on thoracic terga II–III, p_3_ – setae p_3_ on abdominal tergum IV, e/cl – ratio empodium/claw.

Species	d_2_	oc_2_	m_6_	p_3_	e/cl	Distribution	Habitat preferences
*C. granulata* ^1^	−	−	−	+	0.5–0.7	Polish and Slovak Carpathians (Fig. 1)	Cold and humid places in the mountains: mosses in alpine zone, litter and mosses in dwarf mountain pine zone and deep gorges and caves in montane forests zone
*C. lawrencei*	+	+	−	+	0.5–1	Austrian, Italian and Swiss Alps, Apennines, Polish Tatra Mts.	Litter, mosses on rocks in upper montane zone and above, caves
*C. neomeridionalis*	+	+	−	-	0.2–0.3	Slovenian Dinaric Mts, Polish and Ukrainian Carpathians	Litter, mosses on rocks in montane zone
*C. scotica* ^2^	−	−	−	-	0.7–1.1	Belarus, Denmark, Finland, Germany, Great Britain, Ireland, Norway, Poland, Russia, Sweden, Ukraine	Hygrophilous and tyrphophilous species living in lowlands and mountains
*C. silvatica*	+	−	−	-	0.3–0.4	Hungary, Italy, Poland, Romania, Slovakia, Ukraine	Litter, mosses on rocks in upland and mountain forests
*C. stachi* sp. nov.^3^	−	−	+	+	0.4–0.7	Denmark, Germany, Luxembourg, Norway, Poland, Ukraine (Fig. 1)	Litter and mosses in different types of forests in lowlands and mountains, also heathlands and bogs

^1^ Accessory boss near post-antennal organ small (about half the size of posterior lobes of post-antennal organ), mucro narrow at tip (ratio width of apical part/length of mucro = 0.22–0.52 (mean 39). ^2^ Fields of especially coarse granules only on abdominal terga IV–VI. ^3^ Thoracic terga II–III with one additional seta outside lateral sensillum m_7_ present or absent.

#### Ceratophysella granulata

Taxon classificationAnimaliaPoduromorphaHypogastruridae

Stach, 1949

95DCD953-A7CA-50F6-BD37-41C2BF00246E

Ceratophysella granulata Stach 1949: 133

##### Material.

Poland (Carpathians): ISEZ: 2 syntypes on slide (formerly in alcohol), Tatra Mts, Dziura cave, 15.VII.1909, leg. J. Stach; 29 spp. on slides, Tatra Mts, leg. J. Stach; Beskid Mały Mts, male, juv., Zagórze near Skawce, Grota Piaskowa cave, 350 m.a.s.l., XI.1951, leg. Szymczakowski; DIBEC: Tatra Mts (leg. D. Skarżyński): 74 females, 17 males, litter of dwarf mountain pine shrubs on the slopes of the Gładkie Upłaziańskie, at an altitude of 1500–1600 m a.s.l., 13.VII.2001, 14.IX.2002, 18.IX.2004, 14. VIII. 2009; 4 females, 2 males, 2 juv., Chuda Turnia, moss on rocks, 1800 m a.s.l., 19.VIII.2004; 11 females, 5 males, 2 juv., Kraków Gorge, spruce forest litter and mosses on rocks, 1050–1150 m a.s.l., 19.VIII.2004; 4 males, Mylna cave, mosses in the entrance, 1090 m a.s.l., 19.VIII.2004; female, Raptawicka cave, mosses in the entrance, 1150 m a.s.l., 19.VIII.2004; female, juv., Dziura cave, litter in the entrance, 1000 m a.s.l., 24.VIII.1991; 20 females, 15 males, litter of dwarf mountain pine shrubs on the slopes of Sucha Czuba, 1600–1700 m a.s.l., 17.IX.2004; Beskid Żywiecki Mts: 3 females, 5 males, 6 juv., Babia Góra, litter of spruce forest and dwarf mountain pine shrubs, 1300–1500 m a.s.l., 4.VI.1999, leg. A. Smolis; 4 females, 3 males, 5 juv., Pilsko, litter of spruce forest and dwarf mountain pine shrubs, 1300–1500 m a.s.l., 21.IX.2004, leg. D. Skarżyński; Pieniny Mts, 4 females, male, 2 juv., Ociemny valley, 500–600 m a.s.l., mosses on rocks, 26.V.1994, leg. R.J. Pomorski; Beskid Wyspowy Mts, 10 females, male, Zbójecka cave near Limanowa, 900 m a.s.l., bat guano, 12.VII.2007, leg. K. Piksa. Slovakia (Western Carpathians): ISEZ: female, male, Pieniny Mts, Aksamitka cave, VII.1931, leg. Grochmalicki. PJSU: 4 females, 2 males, Veľká Fatra Mts, Horná Túfna cave near Horný Harmanec village, 975 m a.s.l., entrance hall, cave sediment, 26–31.VIII.1999, leg. Ľ. Kováč, 301-99, 302-99; female, Západné Tatry Mts, Brestovská cave near Zuberec village, entrance hall, cave sediment, 22.V.-13.IX.2006, leg. A. Mock, 671-06; 2 males, 3 juv., Belianske Tatry Mts, Kamzíčia jaskyňa cave near Ždiar village, 2002 m a.s.l., 15 m from entrance, cave sediment, 13.IX.1991, leg. Ľ. Kováč; Low Tatras Mts: Demänovská jaskyňa slobody cave near Demänová village, 812 m a.s.l.: female, male, rotten wood, 11.V.2000, leg. P. Ľuptáčik, 84-00; 2 females, male, Mramorové riečisko, bait, 11.V.–27.IX.2000, leg. Ľ. Kováč, 131-00, 133-00; Demänovská ľadová jaskyňa cave, 740 m a.s.l.: 2 females, entrance, talus deposit, 12.V.–28.IX.2000, Ľ. Kováč, 168-00; female, cave entrance, wood, 28.IX.2000, leg. P. Ľuptáčik, 170-00; female, Pustá jaskyňa cave, Hlinená chodba, surface of water puddle, 24.VI.2015, leg. Ľ. Kováč, 82-15; Pieniny Mts, Aksamitka cave near Haligovce village, 756 m a.s.l., leg. Ľ. Kováč: 6 females, 4 males, Blatistý dóm, rotten wood, cave sediment, 12.III.–26.V.1998 , 45-98, 197-98, 198-98, 199-98, 200-98; 2 females, Dóm priekopníkov, bat guano, 26.V.1998, 201-98; 2 females, 1 juv., Dóm priekopníkov, cave sediment, 23.VIII.–7.X.1999, 363-98, 364-98, 366-98; male, soil of herbal cushion in front of cave, 26.V.1998, 202-98; Levočské vrchy Mts, Jaskyňa pod Jankovcom 2 cave near Ľubica village: 3 females, male, Hall A, bat guano, Hall II rotten wood, 5.XI.2010, leg. P. Ľuptáčik, 747-10, 758-10; male, passage B, surface of water puddle, 5.XI.2010, leg. Z. Višňovská, 755-10; Slovak Paradise: Dobšinská ľadová jaskyňa cave near Stratená village, 969 m a.s.l.: female, male, moss on rocks in front of cave, 23.VII.1997, leg. Ľ. Kováč, 126-130-97, 2 females, humus and soil in front of cave, 7.X.2004. leg. V. Šustr, 4 females, male, Psie Diery cave, cave sediment, 6.II.1997, leg. V. Košel, female, Vojenská cave, 20 m from entrance, bait, 6.XII.1998, leg. V. Košel, 22-99, 3 females, cave sediment, 28.I.-6.II.1997, leg. V. Košel, 3 females, Kláštorná cave, cave sediment, 27.I.-4.II.1997, leg. V. Košel, female, 2 males, Duča cave, Dóm, bait, 4.XII.1998, leg. V. Košel, 25-99, male, Stratenská cave, 200 m from entrance, rotten wood, 9.X.1997, leg. Ľ. Kováč, 184-97; male, Muránska planina Plateau, Bobačka cave near Muránska Huta village, 30 m from entrance, cave sediment, 5.X.–9.XI.2000, Ľ. Kováč, 219-00; Čierna hora Mts: 2 females, Veľká ružínska jaskyňa cave near Malá Lodina village, 614 m a.s.l., 100 m from entrance, cave sediment, 10.VIII.–14.X.1996, leg. Ľ. Kováč, 1327-96, male, juv., Malý Ružinok Valley, *Tilio-Acerion*, humus and soil, rotten wood, 19.IX.2009, 23.IV.2010, leg. Ľ. Kováč, 610-09, 139-10; male, Slovak Karst, Šingliarova priepasť cave near Honce village, 680 m a.s.l, 1^st^ Hall, rotten wood, 4.V.2008, leg. P. Ľuptáčik, 215-08.

##### Remarks.

Specificaton of *C. granulata* morphology is provided by Skarżyński (2004a). COI sequences of this species were examined and deposited on BOLD and GenBank by Porco et al. (2012). *Ceratophysella granulata*, with strong tegumentary granulation and distinct fields of coarse granules, resembles *C. stachi* sp. nov. and four other European species of the *C. armata*-group: *C. lawrencei*, *C. neomeridionalis*, *C. scotica*, and *C. silvatica*. Differences between these species are presented in Table 4. The presence of true *C. granulata* is so far confirmed only for the Polish Carpathians (Tatra Mts, Pieniny Mts, Beskid Żywiecki Mts, Beskid Wyspowy Mts, Beskid Mały Mts) and Slovak (Veľká Fatra Mts, Západné Tatry Mts, Belianske Tatry Mts, Low Tatras Mts, Pieniny Mts, Levočské vrchy Mts, Slovak Paradise, Muránska planina Plateau, Čierna hora Mts, Slovak Karst) (Fig. 1), where it inhabits cold and humid places: mosses in the alpine zone, litter and mosses in the dwarf mountain pine zone, deep gorges (litter and mosses), and caves (mosses, litter, and rotten wood at cave the entrance, and bat guano and cave sediments even 100 m from the entrance) in mountain forests zone. At the end of the Pleistocene, this psychro- and hygrophilous species was probably more common in the periglacial region, and due to the warming Holocene climate, its range became limited to scattered, high-mountain refuges and cold caves and other subterranean habitats at lower elevations. Based on the current distribution data, it is concluded that *C. granulata* is endemic to the Western Carpathians. However, to verify this thesis, additional research should be undertaken covering the rest of the Carpathians, the Alps, and other mountainous areas of central Europe.

## Discussion

Traditionally, the most commonly used method in the taxonomy of the genus *Ceratophysella*, as in other Collembola, is the analysis of morphological features (Gisin 1949; Stach 1949; Cassagnau 1959; Yosii 1960; Bourgeois and Cassagnau 1972; Babenko et al. 1994; Jordana et al. 1997; Christiansen and Bellinger 1998; Fjellberg 1998; Thibaud et al. 2004). Recently, hybridization in laboratory conditions and DNA barcoding have also been used, although on a small scale (Skarżyński 2004a, b, c, 2005; Porco et al. 2012; Nakamori 2013). Research on the *Xenylla maritima* complex (Skarżyński et al. 2018) and *Ceratophysella comosa* Nakamori, 2013 showed that combined use of the morphological and genetic criteria may bring good results in establishing species status and support in the family Hypogastruridae. The use of integrative taxonomy methods in this study allowed for the revision of “*C. granulata*” status and the description of a species new to science. Considering its effectiveness and its relative low cost, this method has the potential to bring a significant contribution in the field of taxonomic revision.

## Supplementary Material

XML Treatment for Ceratophysella stachi

XML Treatment for Ceratophysella granulata
